# Self-Powered Sensing System for Electric Vehicle Drive Condition Monitoring and Driving Condition Identification in Intelligent Electric Vehicles

**DOI:** 10.34133/research.1176

**Published:** 2026-03-10

**Authors:** Jianfeng Tang, Haoyuan Li, Yong Hu, Yinglong Shang, Hengyu Li, Hailong Tian, Peng Liu, Liming Zhou, Jianhai Zhang, Hongwei Zhao

**Affiliations:** ^1^ National Key Laboratory of Automotive Chassis Integration and Bionics/School of Mechanical and Aerospace Engineering, Jilin University, Changchun 130025, China.; ^2^ Key Laboratory of CNC Equipment Reliability, Ministry of Education, Changchun 130025, China.; ^3^ Beijing Institute of Nanoenergy and Nanosystems, Chinese Academy of Sciences, Beijing 101400, China.; ^4^ School of Nanoscience and Engineering, University of Chinese Academy of Sciences, Beijing 100049, China.; ^5^ School of Mechanical and Electrical Engineering, China University of Mining and Technology, Xuzhou 221116, China.

## Abstract

It is crucial to monitor the real-time and accurate status of an electric drive system and understand its interaction with driving behavior in order to meet the higher requirements for vehicle safety and reliability in the era of autonomous driving. Traditional wired sensors have limitations in system integration and energy autonomy. This study proposes a triboelectric nanogenerator (TENG) based on the centrifugal-force-enhanced contact mechanism, which can be directly integrated into the transmission shaft of electric vehicles to construct an intelligent self-powered monitoring system. This design effectively overcomes the bottleneck of unstable signals and insufficient durability of traditional rotating TENGs at high speeds by coupling centrifugal force and spring pre-tension and outputs stable and high signal-to-noise ratio sensing signals. On this basis, this study not only achieved high-precision real-time perception of the driving system speed but also further explored the rich information embedded in the centrifugal-force-enhanced contact TENG signal and extended it to the intelligent recognition of driving behavior and road conditions. Based on signal processing and the convolutional neural network and bidirectional long short-term memory model, the system has achieved fault diagnosis of key transmission component bearings in the transmission system with an accuracy rate of up to 96.1%. At the same time, the system can effectively recognize driving behaviors such as sudden acceleration and deceleration (recognition accuracy of 84%), as well as typical road conditions such as flat, slippery, and speed bumps (recognition accuracy of 89.9%), providing key information for automatic driving algorithm calibration and driving safety improvement. The self-powered embedded sensing technology developed in this study provides a new technological path for the efficient energy management and predictive maintenance system of intelligent connected vehicles and is a key sensing node for building future autonomous transportation systems.

## Introduction

Electric vehicles are accelerating the replacement of traditional fuel vehicles and becoming a key force in promoting low-carbon and intelligent transformation in the transportation sector [1–3]. Among them, the electric drive system, as the power core of electric vehicles [4,5], has long faced complex working conditions of high speed and high load [6,7], and its reliability directly determines the performance and driving safety of the entire vehicle [8,9]. However, the cumulative damage and gradual performance degradation characteristics exhibited by electric drive assemblies under complex load spectrum conditions can easily induce transmission function degradation or even catastrophic failures, which has become the core bottleneck restricting their service life [10–12]. In addition, with the rapid development of intelligent connected vehicles and autonomous driving technology, higher requirements have been put forward for the perception and decision-making ability of the entire vehicle. Driving behavior and its interaction with the power system have become key factors affecting vehicle safety and energy efficiency. In addition, the perception of real road conditions under driving conditions, such as speed bumps and slippery roads, as well as the driving status of vehicles, such as rapid acceleration and deceleration, is particularly important. The perception of this information plays a crucial role in achieving advanced autonomous driving functions such as predictive chassis control and adaptive energy management. Therefore, there is an urgent need to establish a real-time, high-precision multiparameter monitoring system that meets the dual requirements of health management for tram drive systems and interactive perception of vehicle environments. By integrating sensing devices to sense the operating status of electric drive system motors [13], real-time analysis of driving interaction behavior can be achieved. By obtaining characteristic signals such as gear meshing status [14] and bearing vibration [15] of the electric drive system, early fault warning and performance optimization of the electric drive system can be achieved. Although monitoring technologies based on current or vibration sensors [16–18] have been partially applied in engineering, they still have limitations, such as dependence on external power supply, signal attenuation caused by complex cables, insufficient adaptability for installation in compact spaces, and deterioration of signal-to-noise ratio in strong electromagnetic interference environments [19,20]. These defects seriously constrain the reliability and universality of monitoring systems and become particularly prominent in intelligent vehicle monitoring that requires distributed, robust, and self-driving perception.

The rise of the triboelectric nanogenerator (TENG) [21–25] provides innovative solutions for the health monitoring of electric drive systems. Based on the coupling effect of frictional charging and electrostatic induction, a TENG, with its self-powering characteristics [26–29], high signal-to-noise ratio sensing capability [30,31], and excellent structural adaptability [32–34], can effectively capture the energy generated by mechanical movements such as vibration and rotation [35–37] and directly convert it into analyzable electrical signals, fundamentally avoiding the dependence of traditional sensors on external power sources and complex wiring [38,39]. In recent years, a series of advances have been made in the application exploration of TENGs in the field of vehicle state perception. For example, a bearing structure TENG achieves speed sensing and energy harvesting simultaneously at a speed of approximately 1,500 rpm through integrated 3-dimensional (3D) printing and polytetrafluoroethylene (PTFE) roller design, demonstrating the potential for structural and functional integration [40]. A rotary switch water-based TENG utilizes the spatial relationship changes between water and electrodes during the rotation process, which can not only collect the mechanical energy of wheel rotation but also detect road slope and wheel speed based on the changes in output signals, expanding the application dimensions of TENGs in vehicle attitude perception [41]. In addition, Lu et al. [42] achieved real-time self-powered monitoring of multidimensional motion states such as vehicle acceleration, angular velocity, and tilt angle by integrating TENGs with electromagnetic generators and introducing a magnetic repulsion adjustment mechanism; Guo et al. [43] achieved continuous and stable monitoring of vehicle speed and braking status over a wide range of vehicle speeds by constructing a frictional constant-current source to provide stable power to Hall elements, demonstrating the feasibility and reliability of combining TENGs with standard vehicle electronic components. These works have promoted the practical exploration of TENGs in vehicle systems from different perspectives. In addition, in terms of transmission system status monitoring, research has preliminarily verified the potential application of TENGs in low-speed scenarios such as gear tooth breakage recognition and bearing pitting detection [44–47]. However, although the above work demonstrates the broad application prospects of TENGs in the field of vehicle sensing, there are still limitations and challenges in applying them to the extreme working conditions of wide speeds and variable loads faced by electric vehicle drive systems. Firstly, most existing TENG designs mainly serve the perception of specific and relatively single physical quantities and have shortcomings in covering the high-speed range of electric drive systems under all operating conditions while maintaining high signal fidelity. More importantly, the centrifugal force generated by high-speed rotation can easily lead to contact instability or gap fluctuations between the rotor and the stator [48]. This not only directly causes output signal attenuation and distortion and introduces complex modulation sideband interference [49] but also fundamentally limits the durability and signal quality of TENGs as a highly reliable sensing source at high speeds. Therefore, in complex dynamic working conditions, how to ensure the dynamic stability of the contact interface from a physical mechanism and achieve accurate fault feature extraction and health status diagnosis based on high-quality signals obtained from it is still a key challenge that urgently needs to be overcome in this field.

To address the above challenges, this paper proposes a centrifugal-force-enhanced contact triboelectric nanogenerator (CFEC-TENG), which achieves comprehensive health monitoring of an electric drive system through self-powered sensing and dynamic contact enhancement architecture. The CFEC-TENG adopts an embedded design and is directly integrated into the output shaft end of the electric drive system of the tram. The core mechanism lies in the dynamic coordination between spring pre-tension and centrifugal force, which converts rotational kinetic energy into a stable interface contact pressure, thus enabling the output of high-fidelity frictional electrical signals that are strictly synchronized with shaft speed even under high-speed conditions. In addition to traditional bearing component fault diagnosis, the rich and high-fidelity signal characteristics output by the CFEC-TENG also enable it to be used for monitoring and analyzing driving behavior (such as rapid acceleration and deceleration) and road conditions (such as flat roads and speed bumps). This provides key data support not only for the health management of the power system but also for the development and calibration of the auto drive system, effectively bridging the perception gap between mechanical status and vehicle operation. Based on a high-precision electric drive dynamic simulation test bench built independently, this study deeply analyzed the output performance of the CFEC-TENG under different load conditions; explored the influence of working conditions, materials, and structural parameters on its output; and verified its robustness under different working conditions. Furthermore, this study analyzed the time–frequency domain characteristics of triboelectric signals in typical fault modes under different load spectra. By combining it with the convolutional neural network and bidirectional long short-term memory (CNN–BiLSTM) model, accurate fault classification of key bearing transmission components was achieved, with a diagnostic accuracy rate of 96%. At the same time, the sensing architecture also demonstrates high efficiency in recognizing and classifying driving patterns and road conditions. Compared with traditional monitoring schemes, the CFEC-TENG avoids the occupation of compact space by complex wiring through passive and self-powered design. Its inherent self-powered characteristics and dynamic anti-interference capability lay the technical foundation for the next generation of intelligent vehicle operation and maintenance systems. This innovation is particularly important for intelligent connected vehicles, as reliable and energy-independent sensing is crucial for achieving higher levels of automation and operational efficiency.

## Results and Discussion

### Structures and working principle of the CFEC-TENG

**Fig. 1.**
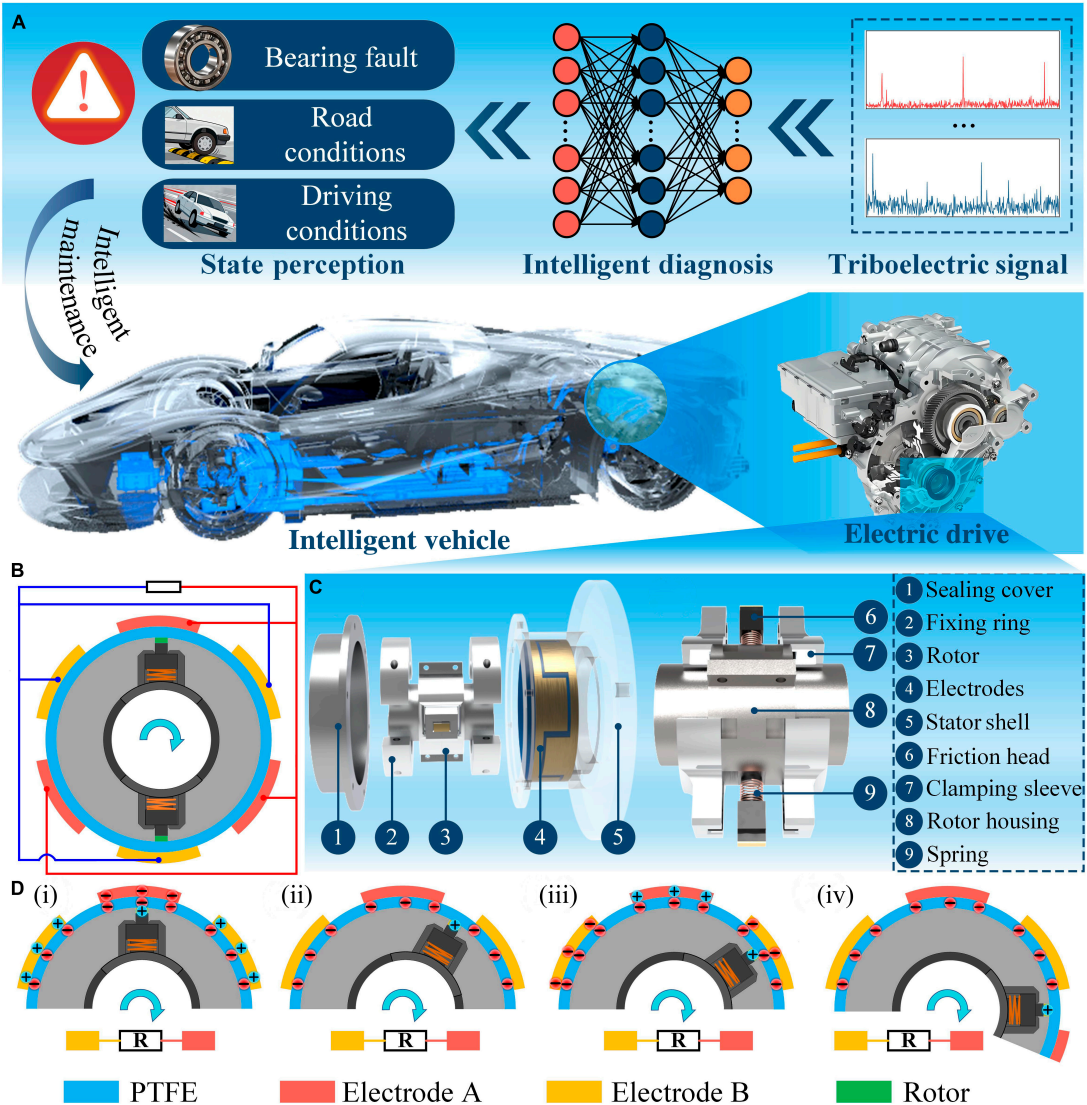
Design and working principle of the centrifugal-force-enhanced contact triboelectric nanogenerator (CFEC-TENG). (A) Application diagram of the CFEC-TENG; (B) structural schematic diagram; (C) main structure; (D) working principle diagram (i) Stage 1; (ii) Stage 2; (iii) Stage 3; (iv) Stage 4. PTFE, polytetrafluoroethylene.

The engineering application of the CFEC-TENG covers the entire closed-loop process from signal perception to intelligent operation and maintenance, as shown in Fig. 1A. This device is embedded and integrated into the key monitoring points of the output shaft of the electric drive system of electric vehicles. By capturing the rotational kinetic energy of the output shaft, it drives the relative friction between the stator and the rotor, generating triboelectric signals that are strictly synchronized with the shaft speed. Combined with the speed ratio relationship of the electric drive gearset, the CFEC-TENG can accurately monitor the rotation status of the motor. The collected raw signals are preprocessed and input into a deep learning neural network. Through feature extraction and pattern recognition, the intelligent diagnostic module can accurately distinguish typical fault conditions of the transmission bearings in the electric drive system and can also analyze and obtain the current road conditions and driving status of the vehicle in real time. By obtaining perceptual information, it can be mapped to the electric drive system to generate targeted driving and maintenance recommendations.

The main structure of the CFEC-TENG is shown in Fig. 1B and C, consisting of a flexible comb-shaped rotor and a stator fixed to the electric drive housing. The stator is covered with forked electrodes inside, and the electrode surface is coated with a layer of PTFE film to optimize charge capture efficiency. The rotor part includes 2 symmetrically distributed arc-shaped comb teeth, with a copper foil covering the top of the comb teeth and a flexible spring mechanism connected to the bottom to reduce friction during rotation. This ensures that the rotor can maintain smooth rotation and output stable electrical signals even when quickly switching between high and low speeds. At the same time, when the speed increases, the rotor comb teeth undergo radial displacement under centrifugal force, markedly increasing the effective contact pressure with the stator triboelectric layer. The contact interface pressure can be dynamically adjusted through centrifugal force. In addition, the rotor is equipped with a sealing cover on the outside to prevent the invasion of oil and dust, ensuring the long-term stable operation of the device under complex working conditions.

Figure 1D illustrates in detail the power generation mechanism of the CFEC-TENG, which is based on the synergistic effect of contact electrification and electrostatic induction. When the comb teeth of the flexible comb-shaped rotor slide on the stator raceway, due to the triboelectric effect between the PTFE film and the copper foil, equal amounts of opposite-numbered charges are generated on the stator finger electrodes and comb teeth surfaces (Fig. 1D (i)). During this process, the PTFE film tends to capture electrons, while the copper foil tends to lose electrons, forming a double layer at the contact interface. As the comb teeth slide from electrode A to electrode B, the electrostatic induction effect drives free electrons to migrate from electrode A to electrode B through an external load (Fig. 1D (ii)). When the comb teeth completely cover electrode B (Fig. 1D (iii)), charge transfer reaches dynamic equilibrium, and the current in the external circuit reaches its maximum value. Subsequently, the comb teeth continue to slide and gradually detach from electrode B, causing free electrons to flow back from electrode B to electrode A through an external load, forming a reverse current (Fig. 1D (iv)). The above 4 stages (i to iv) constitute a complete power generation cycle, and this periodic charge transfer generates a continuous ac signal in the external circuit, whose frequency is linearly related to the rotor speed, providing direct electrical characterization for speed monitoring.

The performance improvement of the CFEC-TENG is due to the CFEC mechanism. To quantitatively describe this phenomenon, we consider the dynamic contact between the triboelectric layer and the comb teeth, establishing the dynamic equation of the rotating system:

$$m\frac{d^{2}x}{dt^{2}}+c\frac{dx}{dt}+k_{\text{elastic}}x^{\frac{3}{2}}=m\omega^{2}\left(t\right)r-F_{\text{preload}}$$ (1)

where *x*(*t*) is the contact compression displacement, *ω*(*t*) is the time-varying angular velocity, and $k_{\text{elastic}}$ is the Hertz contact stiffness.

By solving the response of the system in steady state, the equivalent contact force expression can be obtained:

$$F_{\text{contact}}=F_{\text{preload}}+m\omega^{2}r$$ (2)

This formula indicates that the contact force is contributed by both pre-tension force and centrifugal force, and the centrifugal force increases with the increase in rotational speed.

Based on the Hertz contact theory, the relationship between contact area *A* is

$$A=\pi {\left(\frac{3F_{\text{contact}}R^{*}}{4E^{*}}\right)}^{2/3}$$ (3)

where R^*^ is the equivalent curvature radius and E^*^ is the equivalent elastic modulus.

By combining Eqs. (2) and (3), the variation law of contact area *A* with rotational speed can be obtained:

$$A=\eta {\left(F_{\text{preload}}+m\omega^{2}r\right)}^{2/3}$$ (4)

where $\eta =\pi {\left(\frac{3R^{*}}{4E^{*}}\right)}^{2/3}$.

In order to reveal the microscopic contact mechanism of centrifugal force on surface charge density and quantify the phenomenon of triboelectric charge density increasing with rotational speed, we introduce a surface charge density model:

$$\sigma =\sigma_{0}\;\left[\tanh\left(\alpha \frac{\;P\;}{P_{0}}\right)\right]\cdot e^{-\frac{R_{a}}{R_{a0}}}$$ (5)

where $\sigma_{0}$ is the static contact charge density, $P=F_{\text{contact}}/A$ is the contact pressure, and $R_{a}$ is the surface roughness.

Combining the expression of contact force and contact area, the triboelectric charge density σ can be expressed as

$$\sigma =\sigma_{0}\;\left[\tanh\left(\alpha \frac{\;F_{\text{preload}}+m\omega^{2}r\;}{\pi P_{0}{\left(\frac{3F_{\text{contact}}R^{*}}{4E^{*}}\right)}^{2/3}}\right)\right]\cdot e^{-\frac{R_{a}}{R_{a0}}}\;$$ (6)

This model reveals the nonlinear growth characteristics of charge density σ with rotational speed.

The output voltage and current of the CFEC-TENG increase with the increase in speed. Based on the expression of triboelectric charge density and contact area, the output voltage $V_{\mathrm{OC}}$ can be expressed as

$$V_{\mathrm{OC}}=\frac{\sigma \mathrm{Ad}}{\varepsilon_{0}\varepsilon_{r}\;}=\mu \omega^{4/3}\tanh\left(\beta \omega^{2/3}\right)\;$$ (7)

where *d* is the thickness of the triboelectric layer and $\varepsilon_{0}$ and $\varepsilon_{r}$ are the vacuum dielectric constant and relative dielectric constant, respectively. The short-circuit current $I_{\mathrm{SC}}$ is determined by the charge transfer rate:

$$I_{\mathrm{SC}}=\frac{\sigma A}{T}\cdot \frac{dA}{dt}=\lambda \omega^{7/3}{\mathrm{sech}}^{2}\left(\beta \omega^{2/3}\right)\;$$ (8)

### Electrical characteristics and performance of the CFEC-TENG

**Fig. 2.**
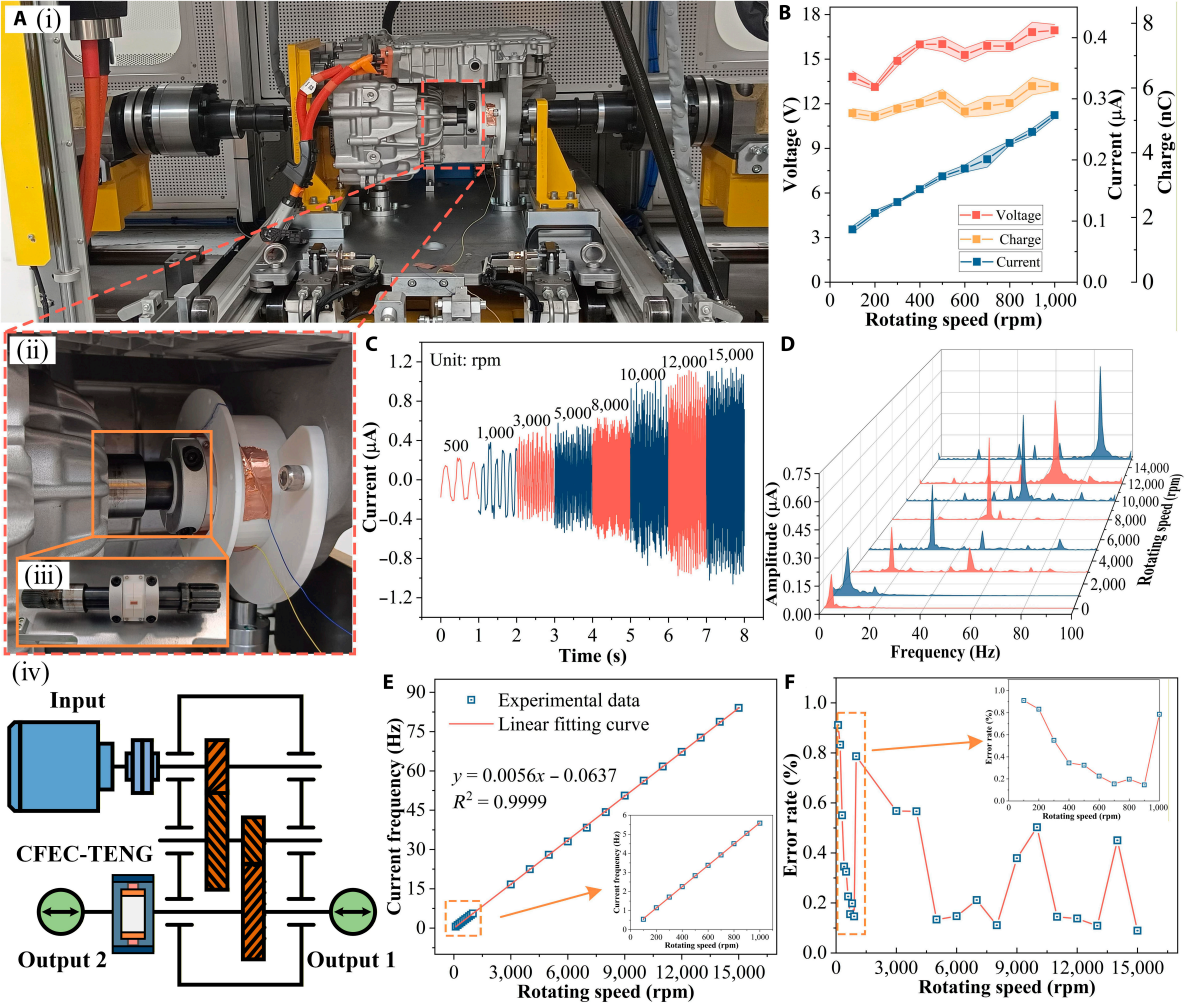
Electrical performance test of the CFEC-TENG. (A) (i) Electric drive system test platform for the CFEC-TENG function test, (ii) installation diagram, (iii) integrated diagram of the rotor and output shaft, and (iv) internal structure diagram of the electric drive reducer; (B) electrical performance test; (C) different speed currents; (D) different speed current frequencies; (E) current frequency and speed fitting curve; (F) error with actual value.

To characterize the performance of the CFEC-TENG at different speeds, we developed an electric drive system testing platform that can accurately simulate the actual working conditions of electric vehicles for experiments. As shown in Fig. 2A (i), the platform is equipped with high-power load motors at both ends, with a maximum output speed of up to 18,000 rpm, and the central platform integrates an actual tram drive system. By precise installation, the CFEC-TENG stator is rigidly fixed to the drive housing (Fig. 2A (ii)), while the rotor is directly coupled to the output shaft (Fig. 2A (iii)). Figure 2A (iv) presents in detail the internal structure of the electric drive reducer, including the spatial arrangement of the input shaft, output shaft, and gearset, with a transmission ratio of 11.88.

By analyzing the electrical performance of the CFEC-TENG in the speed range of 100 to 1,000 rpm (Fig. 2B), it can be clearly observed that the output voltage and transferred charge show a slight upward trend with increasing speed. This phenomenon is mainly attributed to the centrifugal effect, which enhances the contact tightness between the triboelectric layer and the comb teeth, thereby improving the charge transfer efficiency. As the rotational speed increases, the frequency of the comb teeth sliding on the positive and negative poles also increases, which accelerates the transfer of triboelectric charges in the external circuit, resulting in a clear correspondence between peak current and the rotational speed. In this lower speed range, the growth of output is relatively gentle, indicating that the increase in contact force is initially dominated by spring preload, and centrifugal force gradually increases. As the system transitions to the medium-to-high-speed range, the growth of output becomes apparent. Figure 2C shows the output current results of the CFEC-TENG at speeds of 500 to 15,000 rpm. Afterward, by performing Fourier transform on the output current, the frequency spectrum shown in Fig. 2D was obtained. The current growth trend observed from the waveform and spectrum is the result of the synergistic improvement of contact efficiency by centrifugal force and spring preload, which was quantitatively verified in subsequent experiments. Figure 2E shows the corresponding relationship between current frequency and electric driving speed. The results indicate that both have extremely high linearity, *R*^2^ = 0.9999. By comparing and analyzing the frequency fitting curve with the actual speed data, the CFEC-TENG exhibits extremely high-speed measurement accuracy in all tested speed ranges, with a frequency speed conversion error consistently below 1% (Fig. 2F). This further confirms the excellent linear relationship between the signal frequency of the CFEC-TENG and the electric drive speed, indicating that it can be used as an ideal sensing signal.

### The speed-sensing capability of the CFEC-TENG

**Fig. 3.**
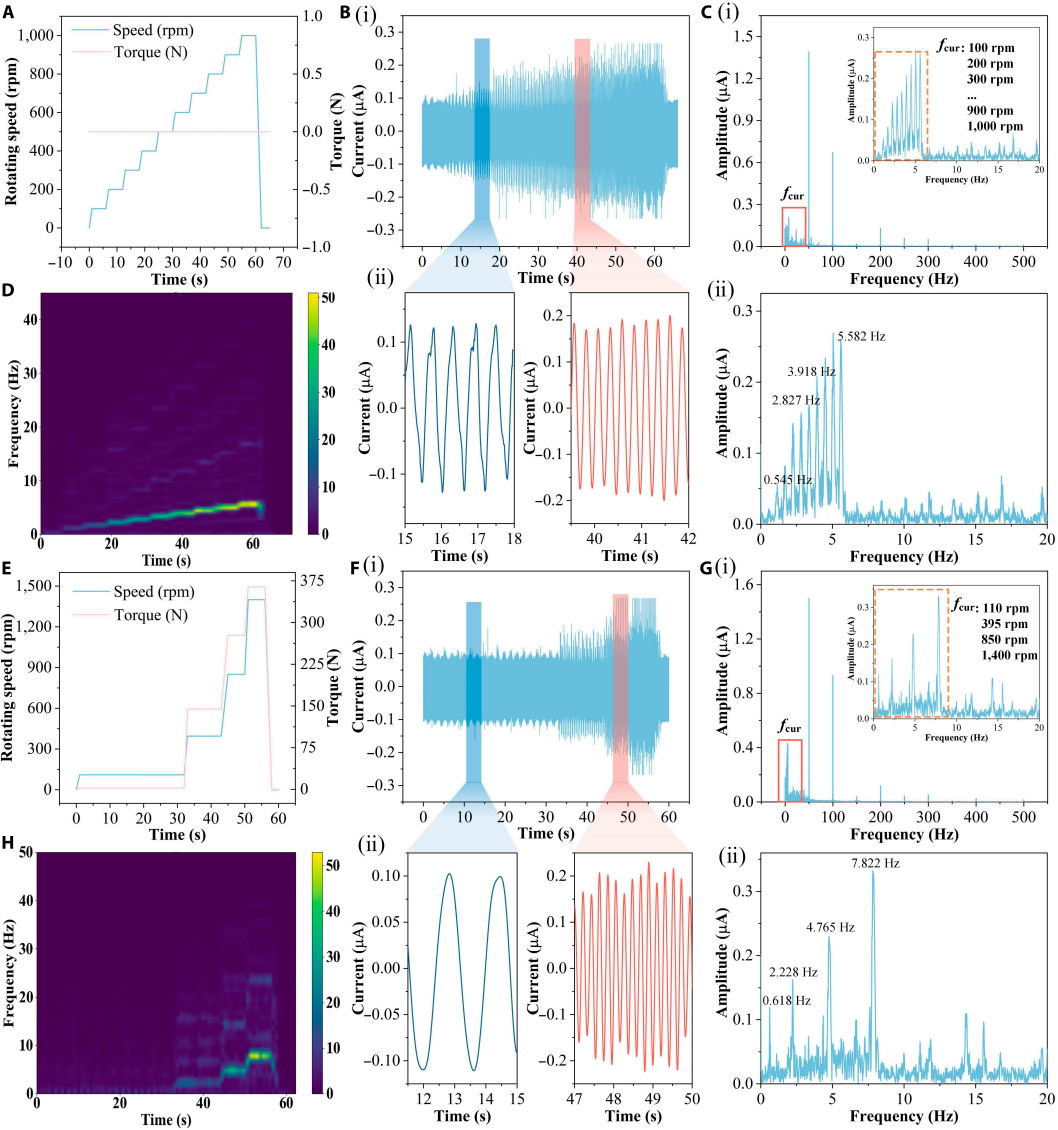
Signal processing of the CFEC-TENG. (A) Input linear load spectrum; (B) (i) corresponding current and (ii) local amplification of current; (C) (i) corresponding spectrum and (ii) localized amplification of the spectrum; (D) time–frequency diagram; (E) random torque and speed composite working condition; (F) (i) corresponding current and (ii) local amplification of current; (G) (i) corresponding spectrum and (ii) spectrum local amplification; (H) time–frequency diagram.

To comprehensively evaluate the sensing performance of the CFEC-TENG under dynamic conditions, we designed 2 different operating conditions: One is a linear increasing operating condition with a speed ranging from 0 to 1,000 rpm, and the other is a combination of random speed and torque. Figure 3A shows the input speed curve of the electric drive system test bench under the first operating condition, where the speed increases linearly with time. The corresponding CFEC-TENG response current is shown in Fig. 3B, and the current amplitude increases with the increase in speed, and the current waveform shows a periodic characteristic highly consistent with the change in speed. By performing a spectral analysis on the response current (Fig. 3C), the frequency components corresponding to each rotational speed can be clearly observed, and there is a strict linear relationship between frequency and rotational speed. This spectrum indicates that the signal energy is concentrated at the fundamental frequency strictly corresponding to the real-time speed, and the spectral lines are sharp with weak background noise. The frequency domain confirms that the sensing response still has good linearity under time-varying conditions. Furthermore, the time–frequency diagram obtained through short-time Fourier transform (STFT) as shown in Fig. 3D clearly demonstrates the continuous and smooth increase in signal frequency components over time, confirming the real-time responsiveness and signal consistency of the sensing system from the joint time–frequency dimension.

To further verify the applicability of the CFEC-TENG under complex operating conditions, we designed a random speed and torque composite operating condition (Fig. 3E). Under this operating condition, the input speed and torque both vary randomly with time, simulating the dynamic load conditions during the actual operation of the electric drive. Figure 3F shows the response current of the CFEC-TENG under this operating condition. Despite the complex input conditions, the current signal can still accurately reflect the speed changes. By performing a spectral analysis on the response current (Fig. 3G), it can be observed that the distribution of each frequency component in the spectrum is highly consistent with the changes in input speed. This spectrum further reveals that under random excitation, the output of the CFEC-TENG can accurately reproduce the instantaneous changes in speed in the frequency domain, and the appearance of each frequency component corresponds closely to the speed, fully demonstrating its excellent dynamic tracking ability and signal fidelity. In addition, the time–frequency characteristics of the current signal were further revealed by generating a time–frequency graph as shown in Fig. 3H through STFT. The results showed that the CFEC-TENG is very sensitive to changes in speed and can track dynamic changes in speed in real time, fully demonstrating its accuracy under complex operating conditions.

### Performance testing of the CFEC-TENG

**Fig. 4.**
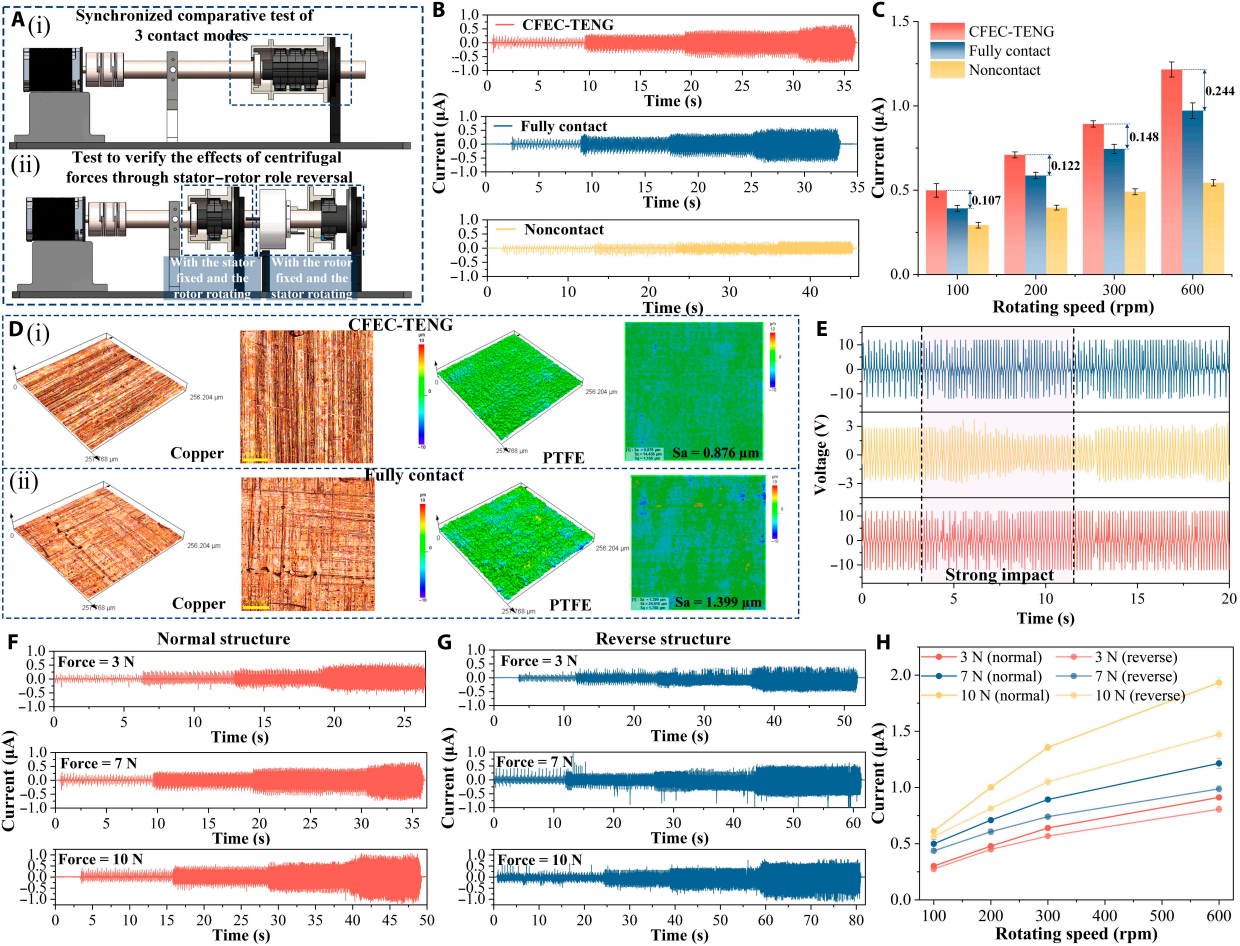
Performance comparison and mechanism verification of the CFEC-TENG. (A) Schematic diagrams of the experimental setups (i) Synchronized comparative test of three contact modes; (ii) Test to verify the effects of centrifugal forces through stator-rotor role reversal; (B) output current waveforms of 3 structures; (C) statistical comparison of output current amplitudes; (D) surface morphology of the tribo-pairs after operation (i) Surface morphology of CFEC-TENG; (ii) Surface morphology of fully contacted TENG; (E) signal responses under sudden mechanical impact; (F) current waveforms of normal and reverse structures; (G) current–speed curves of normal and reverse structures; (H) current versus rotational speed for normal and reverse structures under different spring preload forces.

To verify the comprehensive advantages of the CFEC-TENG in terms of contact mechanism, output performance, durability, and robustness and to reveal its principle of the CFEC, we designed a control experiment as shown in Fig. 4A. Figure 4A (i) shows the synchronous comparative testing platform of the CFEC-TENG with a fully contact TENG and a noncontact TENG. It is used to horizontally evaluate the electrical output and stability performance of different contact mechanisms under the same operating conditions, thereby directly highlighting the overall advantages of elastic contact design. Figure S1 is a detailed comparison of the 3 structures. To further quantitatively reveal the independent effect of centrifugal force, we designed a stator rotor role reversal experiment as shown in Fig. 4A (ii). The reverse structure has exactly the same sliding frequency as the normal structure, but centrifugal force is stripped away from the rotor spring contact system. Therefore, quantitative verification of CFEC has been achieved.

Unlike the complex drive bench used to simulate the actual dynamic loads of electric vehicles in the sections “Electrical characteristics and performance of the CFEC-TENG” and “The speed-sensing capability of the CFEC-TENG”, the experimental platform specially built in this section for fair comparison between structures adopts a more simplified design. This platform removes the actual electric drive system and its complex transmission mechanisms such as gearboxes, which can avoid interference and focus on the mechanical and electrical characteristics’ differences of different TENG structures themselves. It should be noted that due to the omission of the gearbox, the CFEC-TENG is directly integrated into the output shaft of the drive motor in this comparison platform, and its operating speed is strictly consistent with the motor speed, which is different from the shaft end speed obtained through gearset shifting in the original complex test bench.

Afterward, we tested the electrical output performance of the 3 structures at 100, 200, 300, and 600 rpm, respectively. The waveform comparison results are shown in Fig. 4B, and the output current statistical analysis results are shown in Fig. 4C. The results showed that the output currents of all 3 increased with the increase in speed, but the current amplitude and slope of the CFEC-TENG with the increase in speed were substantially higher than those of the fully contact TENG and noncontact TENG structures. For example, at 100 rpm, its output current is about 0.107 μA higher than that of the fully contact structure, and this difference further widens with increasing speed. At 600 rpm, its output current is about 0.244 μA higher than that of the fully contact structure. This directly proves that the contact efficiency of the CFEC-TENG is further enhanced with increasing rotational speed; not only does it have a higher output capability, but also its energy conversion efficiency increases faster with increasing rotational speed.

Afterward, we conducted a systematic comparison of the friction pair surfaces after 3 sets of experiments, and all 3 structures were operated synchronously at 600 rpm for 2 h. The macroscopic observation results shown in Fig. S2 indicate that the noncontact TENG, due to the lack of physical contact, maintains its friction pair surface in its initial state with no obvious wear marks. However, the surface of the traditional fully contact TENG shows visible scratches to the naked eye, with dark wear products attached to the copper foil locally. The PTFE film surface even shows slight signs of rupture, indicating severe material damage. To accurately quantify the degree of wear, we further used laser confocal microscopy to characterize the microstructure of the friction interface between the CFEC-TENG and the fully contact TENG. As shown in Fig. 4D, after the same operating time, the surface of the copper foil with rigid contact structure is covered with deep and disorderly plow grooves. The arithmetic mean roughness (Sa) of its PTFE film is as high as 1.399 μm, and the 3D morphology presents locally steep valleys and protrusions, indicating that it has undergone nonuniform severe wear. In contrast, the copper foil surface of the CFEC-TENG has shallow scratches only, and the roughness of its PTFE film is only 0.976 μm, with a highly uniform distribution of morphology. This comparison intuitively verifies that the spring preloading and flexible contact design of the CFEC-TENG structure effectively homogenizes the contact stress, thereby transforming the wear mechanism of the contact TENG from destructive severe wear to mild progressive wear.

In order to more intuitively verify the robustness advantage of the CFEC-TENG under dynamic conditions, we introduced instantaneous severe shaking and impact into the shell with 3 types of TENG structures installed, as shown in Fig. S3. The output signals of the 3 structures were synchronously collected and compared, and the results are shown in Fig. 4E. Analysis shows that under sudden impacts, noncontact structures experience marked signal amplitude disturbances and waveform distortions due to drastic changes in air gaps. In contrast, the CFEC-TENG, with its spring preloaded elastic contact mechanism, can still maintain stable contact at the interface under impact. Its output signal produces only a momentary fluctuation with extremely small amplitude and duration and then quickly returns to a stable waveform.

To strictly distinguish and quantitatively characterize the dynamic contact mechanism formed by the comb-shaped structure and spring preloading, which is enhanced by centrifugal force and independent of the sliding frequency contribution, we designed a stator rotor role reversal control experiment as shown in Fig. 4A (ii). In the inverted structure, by rotating the stator, we ensure that its sliding frequency is exactly the same as that of the normal structure. At this point, the comblike structure on the rotor and the spring preloading system are kept stationary, thereby separating the dynamic effect of centrifugal force on the rotor comb teeth and spring system from the entire contact mechanics.

We first compared the frictional electrical signals of 2 structures under 3 preload forces of 3, 7, and 10 N at 4 speeds of 100, 200, 300, and 600 rpm. The collected signal waveforms are shown in Fig. 4F and G. It can be intuitively observed that the current amplitude of both structures increases with the increase in rotational speed, but the signal waveform output by the normal structure is more stable and regular than that of the inverted structure. On this basis, we conducted multiple repeated measurements and statistical analyses and presented them in the form of bar charts in Fig. S4. The comparison showed that the current of both structures increased with the increase in spring preload force. However, under all test conditions, the current amplitude of the normal structure is always higher than that of the inverted structure. This preliminarily proves that under the condition of strictly consistent sliding frequency, the presence of centrifugal force directly enhances the charge transfer amount of a single sliding event. To further quantitatively reveal its inherent laws, we compared and analyzed the detailed curves of current variation with rotational speed for 2 structures under different spring preload forces, as shown in Fig. 4H. From the comparison curve graph, it can be seen that the slope of the current of the reverse structure with increasing speed remains almost the same under different preload forces. This is because its performance improvement relies entirely on an increase in sliding frequency, and once it is freed from the dynamic assistance of centrifugal force, its growth is determined by the static structure. In sharp contrast, the growth slope of the normal structure of the CFEC-TENG increases with the increase in preload force, and under a 3-N preload force, its slope is about 15% higher than that of the inverted structure. At 7 N, the advantage expands to 31%. At 10 N, the advantage further reaches 45%. The law that the slope of this current increases monotonically with the increase in speed and preload force has good persuasiveness in explaining the enhanced contact of centrifugal force. Therefore, through the quantitative analysis of the above reversal experiment, we can clarify that in the CFEC-TENG, the dominant contribution mechanism of the output growth brought about by the increase in rotational speed is not only the increase in sliding frequency but also the dynamic contact stability enhancement achieved by the synergistic effect of centrifugal force and spring preload force on the comb tooth elastic structure.

**Fig. 5.**
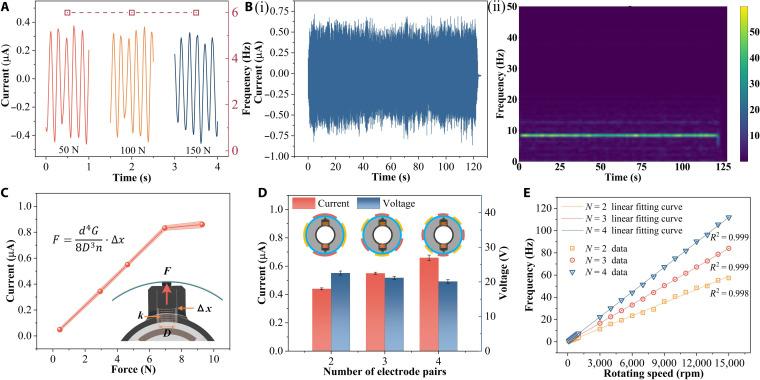
Performance testing of the CFEC-TENG. (A) Different torques; (B) (i) durability testing and (ii) time–frequency diagram; (C) different pretightening forces; (D) different electrode pairs; (E) the fitting of different electrode pairs.

To verify the performance of the CFEC-TENG under different operating conditions, Fig. 5A shows its response characteristics under variable torque conditions. The experimental results show that although the output current amplitude fluctuates slightly with the change in speed, the current frequency exhibits extremely high stability and is not affected by torque changes. Subsequently, we conducted multiple running tests at 2,000 rpm for 5 consecutive hours. Figure 5B (i) shows the electrical output response of the CFEC-TENG after a cumulative 50-h long-term life test. Although there is a certain degree of fluctuation in the current amplitude, it can be seen from the time–frequency plot of the output current (Fig. 5B (ii)) that the current frequency remains stable, indicating that the CFEC-TENG can still provide stable speed sensing after long-term operation.

To clarify the physical process of interface wear between the stator PTFE film and the rotor copper foil of the CFEC-TENG friction pair materials before and after operation, we characterized the 2 friction pair materials using laser confocal microscopy. As shown in Fig. S5, the surfaces of the new copper foil and PTFE film are flat and uniform, and the arithmetic mean roughness (Sa) of the PTFE film is about 0.39 μm. After continuous operation for 30 h, scratches distributed along the sliding direction began to form on the surface of the copper foil, and the height fluctuation of the PTFE film surface slightly increased. The surface roughness of the PTFE film increased to 1.156 μm. After completing a total of 50 h of testing, the scratches on the copper foil surface deepened but remained uniform overall. The roughness of the PTFE film gradually increased to 1.302 μm, and its 3D morphology showed that the surface height distribution was overall uniform, without severe local depressions or material peeling or other destructive features. This indicates that under the structure of the CFEC-TENG, the wear of the friction pair is a gradual and controllable process, and the wear mechanism is mainly uniform and slight wear, rather than severe fatigue or tearing, so the interface state remains stable.

After verifying the reliability of the CFEC-TENG in complex environments, we conducted a systematic research on key parameters to further improve its performance and optimize its performance in practical applications. We studied the output performance of the CFEC-TENG under different preloading forces by changing the spring parameters, as shown in Fig. 5C. The results indicate that as the preclamping force increases, the output current also increases. However, when a certain preclamping force is reached, the output current tends to saturate and no longer continues to increase. The influence of electrode pairs on output performance is shown in Fig. 5D. As the number of electrode pairs increases, the output voltage slightly decreases, mainly due to the decrease in the area of a single electrode as the number of electrodes increases. However, the output current slightly increases with the increase in electrode pairs due to the improvement of charge separation efficiency. Figure 5E shows the linear correspondence between current frequency and rotational speed under different electrode pairs, indicating that the CFEC-TENG can maintain good response capability even with changes in electrode pairs.

### Fault diagnosis capability of the CFEC-TENG

**Fig. 6.**
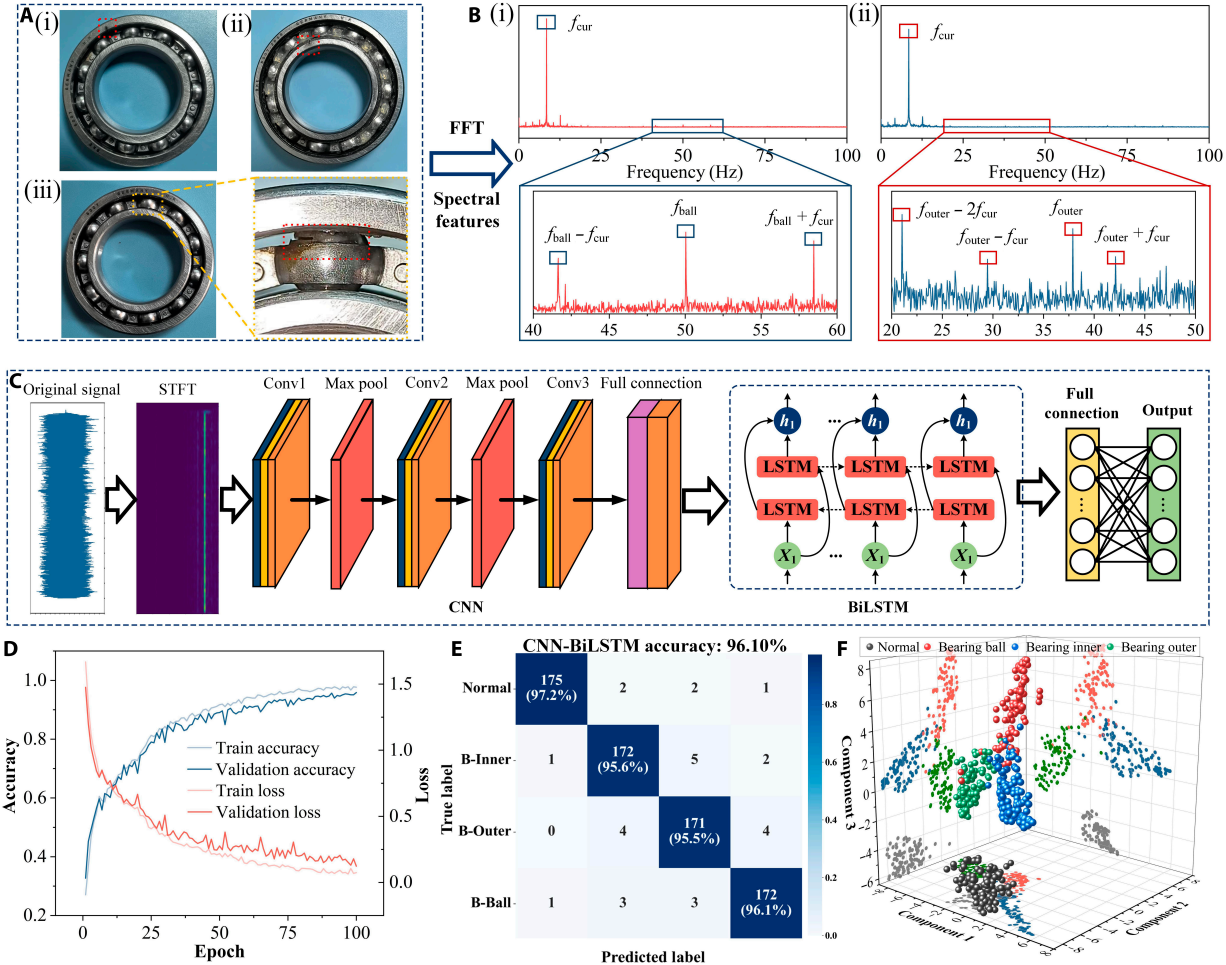
Fault diagnosis capability of the CFEC-TENG. (A) Introduction of a physical image of the malfunction (i) Bearing outer race fault; (ii) Bearing inner race fault; (iii) Bearing rolling element fault; (B) spectrum corresponding to the fault (i) Rolling element fault spectrum; (ii) Outer race fault spectrum; (C) neural network architecture; (D) the training process of data; (E) confusion matrix; (F) t-distributed stochastic neighbor embedding (t-SNE) visualization. CNN, convolutional neural network; LSTM, long short-term memory; BiLSTM, bidirectional long short-term memory; STFT, short-time Fourier transform.

In order to verify the effectiveness of the CFEC-TENG in the diagnosis of transmission bearing faults in electric vehicle electric drive systems, this study artificially introduced 3 typical fault modes of bearing components through wire cutting, including the inner ring, outer ring, and rolling element faults of bearing components, as shown in Fig. 6A. Figure 6B shows the frequency spectrum analysis results of the CFEC-TENG current signal under fault conditions. Compared to a healthy state, local faults can cause fluctuations in triboelectric signals, resulting in substantial changes in the spectral characteristics of the CFEC-TENG output current. For example, faults in the inner and outer rings of a bearing will result in pronounced modulation sidebands at their fault frequencies.

To achieve efficient and accurate fault diagnosis, this study developed an intelligent diagnostic model that combines signal processing and hybrid deep learning architecture, as shown in Fig. 6C. The process first uses STFT to convert the raw triboelectric signal collected by the CFEC-TENG into a 2-dimensional time–frequency map. This time–frequency representation can simultaneously capture the time-domain and frequency-domain characteristics of the signal and serve as input for the neural network. This model uses a convolutional neural network module composed of 3 consecutive convolutional layers. Firstly, the spatial features are automatically extracted from the time–frequency graph. Subsequently, these features are sent to BiLSTM to effectively capture temporal dependencies and contextual information in the feature sequence. This CNN–BiLSTM hybrid architecture fully utilizes the dual advantages of spatial feature extraction and time-series modeling, thereby achieving a more robust and accurate diagnosis. Finally, the learned features were mapped to the corresponding fault categories through a fully connected layer, successfully achieving high-precision fault diagnosis of key transmission components.

In terms of dataset construction, all data come from the electric vehicle drive system test bench integrated with the CFEC-TENG. We collected raw signals of bearings in 4 states: healthy, inner ring fault, outer ring fault, and rolling element fault. Each state was sampled for 300 s at different reference speeds with a sampling frequency of 10 kHz. All raw signals undergo unified denoising and standardization preprocessing, and sample segmentation is performed through a sliding time window (window length of 1 s, overlap of 50%), ultimately constructing a balanced dataset. During the model training phase, we randomly divided the total sample into training and testing sets in a 7:3 ratio and used grid search to systematically optimize the hyperparameters of the CNN–BiLSTM model, such as convolution kernel dimension, number of long short-term memory (LSTM) units, dropout rate, and learning rate, in order to minimize human bias to the greatest extent possible. The experimental results show that the average recognition accuracy of CNN–BiLSTM for 3 types of fault modes is 96.1%. Figure 6D shows the training process. Figure 6E shows the confusion matrix of the diagnostic results. Through t-distributed stochastic neighbor embedding dimensionality reduction visualization (Fig. 6F), the features extracted by CNN–BiLSTM exhibit obvious clustering phenomena in low-dimensional space. This result validates the effectiveness of CNN–BiLSTM in fault feature extraction and the unique advantages of the CFEC-TENG signal in fault diagnosis. At the same time, it also indicates that the CFEC-TENG combined with CNN–BiLSTM scheme has the advantages of high sensitivity, strong anti-interference ability, and low complexity. It can capture weak speed fluctuations and fault impacts and maintain stable signal quality under complex working conditions.

To verify the generalization ability of the model, we designed a cross-validation strategy as shown in Table 1. Strategy 1 is to use data at 1,500 and 3,000 rpm as the training set and data at 4,500 and 6,000 rpm as the testing set. Strategy 2 is the opposite, training with data at 4,500 and 6,000 rpm and testing on data at 1,500 and 3,000 rpm; strategy 3 is to mix the data tested at 1,500 and 3,000 rpm and randomly divide the training and testing sets in a conventional 7:3 ratio.

**Table 1.** Cross-validation strategy

Number	Training set	Testing set
1	1,500 rpm, 3,000 rpm	4,500 rpm, 6,000 rpm
2	4,500 rpm, 6,000 rpm	1,500 rpm, 3,000 rpm
3	(1,500 rpm, 3,000 rpm) × 70%	(1,500 rpm, 3,000 rpm) × 30%

The confusion matrix results shown in Fig. S6 indicate that in strategy 1, the model achieved an accuracy of 88.6%. In strategy 2, the accuracy rate is 86.9%. In strategy 3, the accuracy rate is 93.9%. It is worth noting that in the confusion matrices of strategy 1 and strategy 2, the model maintained high recognition accuracy for the vast majority of fault categories, with the main errors concentrated between a few fault types with similar features. To further objectively evaluate the performance of the CNN–BiLSTM architecture, we compared it with standard CNN, CNN–ResNet, and CNN–Transformer models. All models use the same dataset and training settings. The validation loss curve shown in Fig. S7 shows that the loss curve of CNN–BiLSTM converges the smoothest and fastest, with the lowest validation loss, indicating that it can more effectively capture the temporal dependence of fault signals without obvious fluctuations or overfitting signs. In contrast, the loss curve of the standard CNN model converges slowly and oscillates slightly in the later stage; the CNN–Transformer model also exhibits training volatility. The comparative results indicate that the BiLSTM layer has unique advantages in capturing the inherent temporal dependencies in fault signals, resulting in a more optimal and stable learning process. Figure S8 shows the confusion matrix results. On the independent test set, the overall accuracy of CNN–BiLSTM is substantially better than those of other models. To evaluate the repeatability and reliability of the model, we conducted a repeatability test on all compared architectures, including CNN–BiLSTM. Each model architecture was independently trained 20 times under identical hyperparameters and data-partitioning conditions, with different random seeds initialized each time. The statistical analysis results shown in Fig. S9 indicate that the CNN–BiLSTM model has the highest average accuracy, with accuracy consistently concentrated in the range of 94.5% to 97.6%, which is superior to those of other control models.

### Application of the CFEC-TENG in real vehicle monitoring

**Fig. 7.**
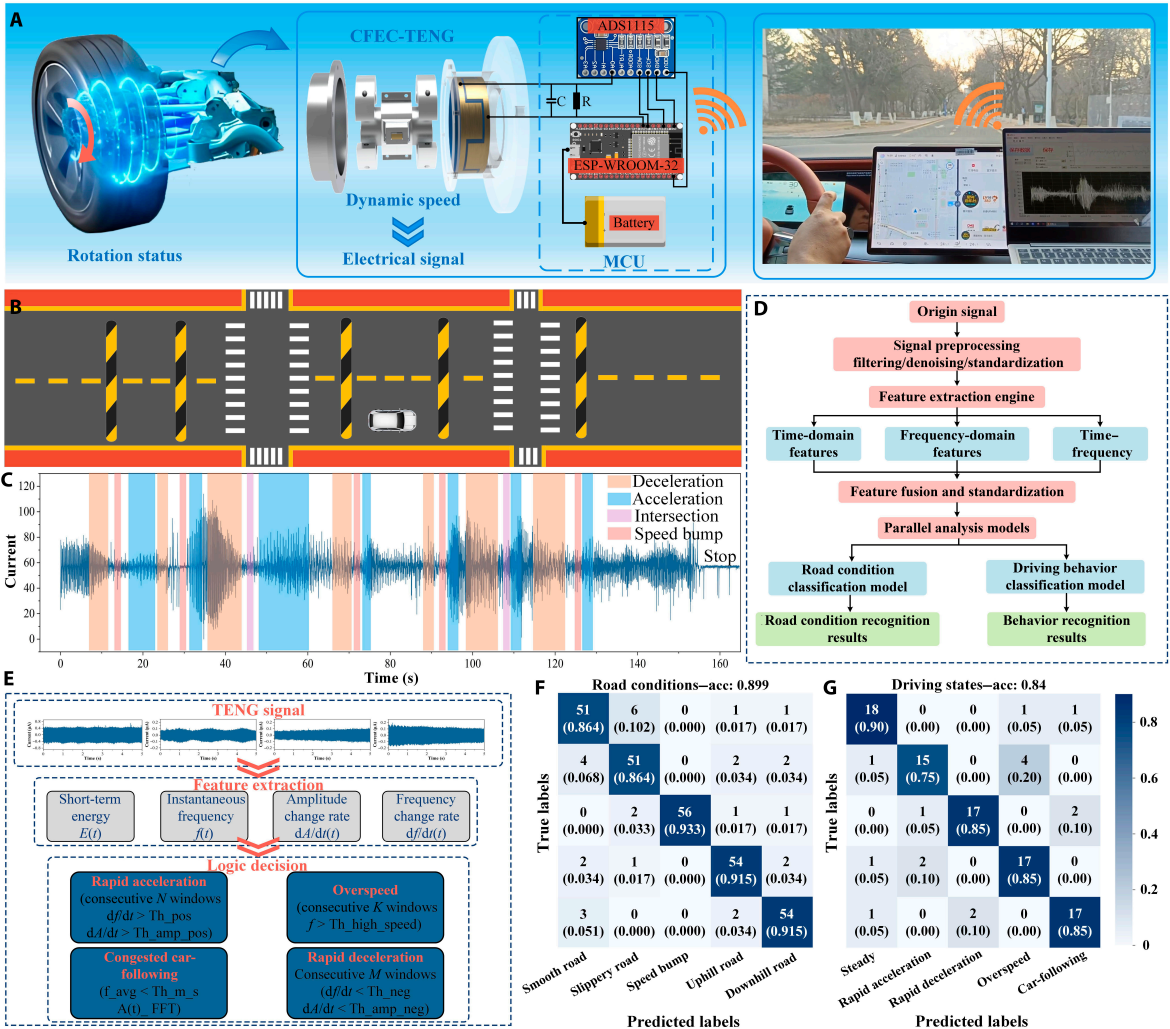
Application testing of the CFEC-TENG in real vehicles. (A) Schematic diagram of signal transmission; (B) measurement of the road condition map; (C) corresponding test result chart; (D) identification method flowchart; (E) judgment logic; (F) road condition confusion matrix; (G) driving state confusion matrix. MCU, microcontroller unit.

To achieve the engineering application of the CFEC-TENG in real vehicle electric drive status monitoring, as shown in Fig. 7A, this work integrates the CFEC-TENG with the drive shaft of the electric drive through embedded packaging. Through its microcontroller unit module, the operating status of the electric drive can be wirelessly transmitted to the upper computer for real-time monitoring.

The test road for the CFEC-TENG is shown in Fig. 7B, which includes main roads, areas with dense speed bumps, and multiphase traffic intersections. During the testing process, the CFEC-TENG exhibited excellent signal fidelity by accelerating and decelerating according to actual road conditions, as shown in Fig. 7C. The output current waveform showed characteristics in the time domain, with a noticeable decrease in current when the vehicle passed through intersections and speed bumps, while the acceleration process responded to an increase in current, meeting the real-time monitoring requirements for vehicle health monitoring systems.

On the basis of basic signal acquisition, a comprehensive analysis framework for advanced perception tasks was further constructed. Figure 7D shows the complete workflow of road condition and driving behavior recognition, including signal preprocessing, multidomain feature extraction (covering time domain, frequency domain, and time–frequency features), feature fusion, and finally classification decisions implemented through parallel analysis models. Specifically, regarding driving behavior recognition, Fig. 7E provides a detailed description of its logical judgment process. Firstly, key features such as instantaneous amplitude, frequency, and their derivatives are extracted from the preprocessed signal. After buffering through a temporal window, a series of logic rules based on specific states are applied to robustly identify 5 types of behaviors: rapid acceleration, rapid deceleration, overspeed, car-following, and steady driving. The effectiveness of this analytical framework is quantitatively validated in Fig. 7F and G. The confusion matrix in Fig. 7F shows the performance of the road condition recognition model in identifying 5 road conditions, smooth road, slippery road, speed bump, uphill road, and downhill road, with an overall accuracy of 89.9%. Figure 7G presents the confusion matrix for driving behavior recognition, with an overall accuracy of 84%. These results fully prove that the CFEC-TENG, in combination with the proposed analysis framework, has successfully exceeded the traditional health monitoring category and can simultaneously realize the intelligent perception of vehicle environment interaction and driver operation, providing a functional sensing solution for the next generation of auto drive system.

## Discussion

The core innovation of the TENG based on the CFEC-TENG proposed in this study lies in the dynamic contact stabilization mechanism that combines spring pre-tension and centrifugal force, effectively solving the long-standing technical bottleneck of signal attenuation caused by contact instability in rotating friction nanogenerators under high-speed conditions. Theoretical analysis shows that this mechanism can transform the unfavorable interference factors in the traditional design of rotational centrifugal force into positive forces that enhance frictional interface contact. The experimental observation results of the system, including the independent contribution of centrifugal force revealed by the reverse control experiment, the comparative verification with noncontact and fully contact structures, the extremely high linearity (*R*^2^ = 0.9999) maintained between current frequency and speed in a wide range of up to 15,000 rpm, the uniform slight wear morphology exhibited by the friction pair after long-term operation, and the rapid recovery ability of the signal under sudden impact, all jointly verified the effectiveness of this theoretical mechanism from multiple dimensions. In addition, the CFEC-TENG also demonstrates the following comprehensive advantages: Firstly, the signal frequency and speed maintain a strictly linear relationship throughout the entire operating range, providing a reliable foundation for high-precision speed tracking; the elastic contact mechanism substantially reduces interface wear and endows the device with excellent resistance to mechanical impact and load fluctuations; and, at the same time, the high-fidelity time–frequency signals output by the system have the potential for multitasking perception, which can support intelligent fault diagnosis of the core bearings of the driving system (accuracy rate of 96.1%) and can also be extended to recognize typical driving behaviors (recognition accuracy rate of 84%) and complex road conditions (recognition accuracy rate of 89.9%). We also recognize the current limitations of this technology, and this study is mainly based on a laboratory bench environment for verification. Although preliminary real vehicle scenario testing has been conducted, the long-term reliability under extremely high and low temperatures, continuous vibration, and complex electromagnetic interference environments still needs to be systematically assessed. In addition, moving from laboratory prototypes to large-scale industrial applications requires further addressing the issues of process consistency, cost control, and engineering integration with existing vehicle electronic and electrical architectures in batch manufacturing. Future work will focus on the comprehensive improvement of environmental adaptability, integration with standard protocols for in-vehicle networks, and exploration of extending its perception framework to a wider range of rotating machinery system status monitoring, thereby promoting the in-depth application of self-powered intelligent sensing technology in high-end equipment and intelligent transportation fields.

## Materials and Methods

### Fabrication of the CFEC-TENG

The flexible combed-tooth rotor and stator of the CFEC-TENG are manufactured by photocuring 3D printing. The inside of the stator is covered with a 0.1-mm-thick forked finger electrode that is 20 mm wide, and the surface of the electrode is coated with 0.05-mm-thick PTFE film. The rotor section consists of a pair of symmetrically arranged arc comb teeth, the top of which is covered with copper foil, and the bottom is connected to a flexible spring mechanism. The outer part of the rotor is equipped with a sealing cover to prevent the ingress of oil and dust.

### Performance characterization

The rotary test bench is jointly developed by Jilin University and First Automotive Works Group, with a precision of ±0.5% full scale and a maximum rotating speed of 18,000 rpm. The corresponding output performance of the CFEC-TENG was tested by an electrometer (Keithley 6514; Tektronix, USA).

## Data Availability

The data that support the findings of this study are available from the corresponding authors upon reasonable request.
