# Supplementary material for: Self-Powered Sensing System for Electric Vehicle Drive Condition Monitoring and Driving Condition Identification in Intelligent Electric Vehicles
Source: Research (Wash D C). 2026 Mar 10;9:1176. doi: 10.34133/research.1176 (PMC12972506; doi:10.34133/research.1176)
Supplement: Supplementary 1 — Figs. S1 to S9 Movies S1 to S3 [file research.1176.f1.zip › Supplementary Materials.docx]

Self-Powered Sensing System for EV-Drive Condition Monitoring and Driving Condition Identification in Intelligent EVs

Jianfeng Tang ^a, b^, Haoyuan Li ^a, b^, Yong Hu ^a, b, *^, Yinglong Shang ^a, b^, Hengyu Li ^d, e, *^, Hailong Tian ^a, b^, Peng Liu ^a, b^, Liming Zhou ^a, b^, Jianhai Zhang ^a, b, *^, Hongwei Zhao ^a, b, c^

*^a^ National Key Laboratory of Automotive Chassis Integration and Bionics/School of Mechanical and Aerospace Engineering, Jilin University, Changchun, 130025, China*

*^b^ Key Laboratory of CNC Equipment Reliability, Ministry of Education, Changchun, 130025, China*

*^c^ School of Mechanical and Electrical Engineering, China University of Mining and Technology, Xuzhou 221116, China*

*^d^ Beijing Institute of Nanoenergy and Nanosystems, Chinese Academy of Sciences, Beijing 101400, China.*

*^e^ School of Nanoscience and Engineering, University of Chinese Academy of Sciences, Beijing 100049, China.*

* Corresponding authors.

Address correspondence to [huyong@jlu.edu.cn](mailto:huyong@jlu.edu.cn) (Y. Hu); lihengyu@binn.cas.cn (H. Li); [jianhaizhang@jlu.edu.cn](mailto:jianhaizhang@jlu.edu.cn) (J. Zhang)

The file includes:

**Figure S1.** Comparison Details

**Figure S2.** Comparison of Wear Conditions

**Figure S3.** Simulating Strong Impact

**Figure S4.** Comparison of current amplitude

**Figure S5.** Surface morphology of PTFE and copper foil at different wear stages

**Figure S6.** Cross validation confusion matrix comparison

**Figure S7.** Comparison of Verification Losses

**Figure S8.** Comparison of confusion matrices for different models

**Figure S9.** Comparison of Average Accuracy

**Movie S1.** CFEC-TENG Simulation of Real Vehicle Operating Conditions on an Electric Drive Test Bench: Condition 1

**Movie S2.** CFEC-TENG Simulation of Real Vehicle Operating Conditions on an Electric Drive Test Bench: Condition 2

**Movie S3.** Installation of CFEC-TENG on an Electric Vehicle for On-Road Testing


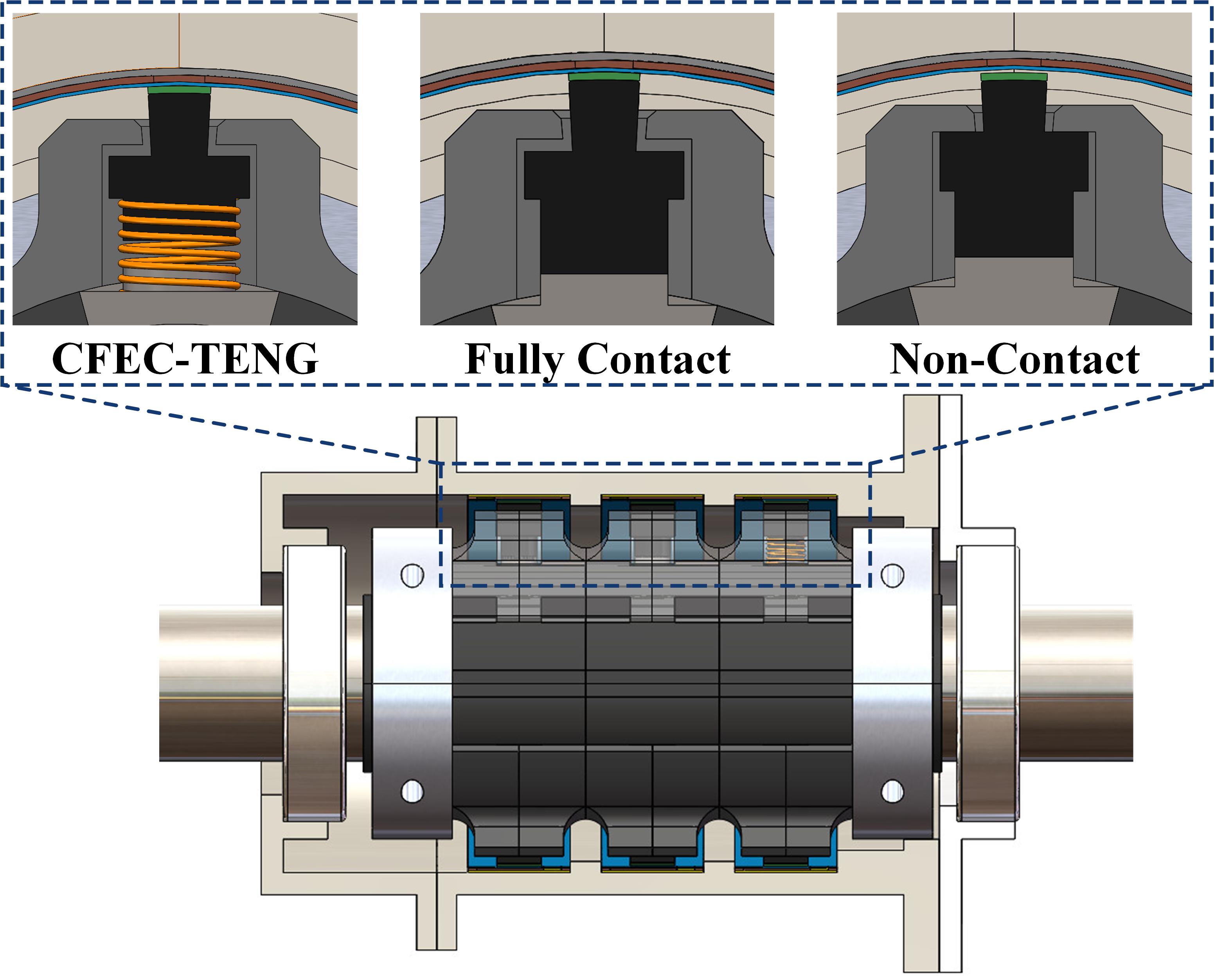


**Figure S1.** Comparison Details


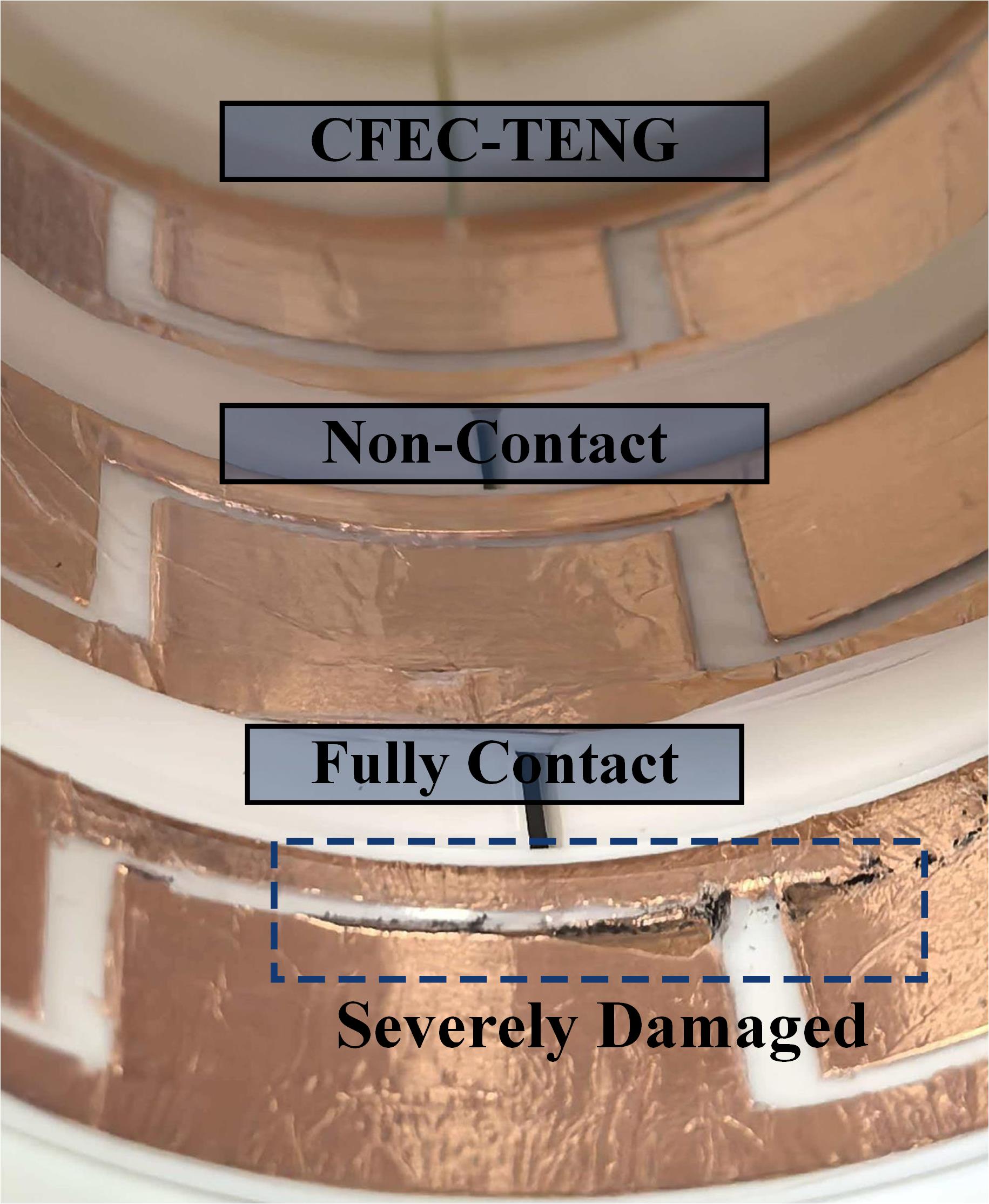


**Figure S2.** Comparison of Wear Conditions


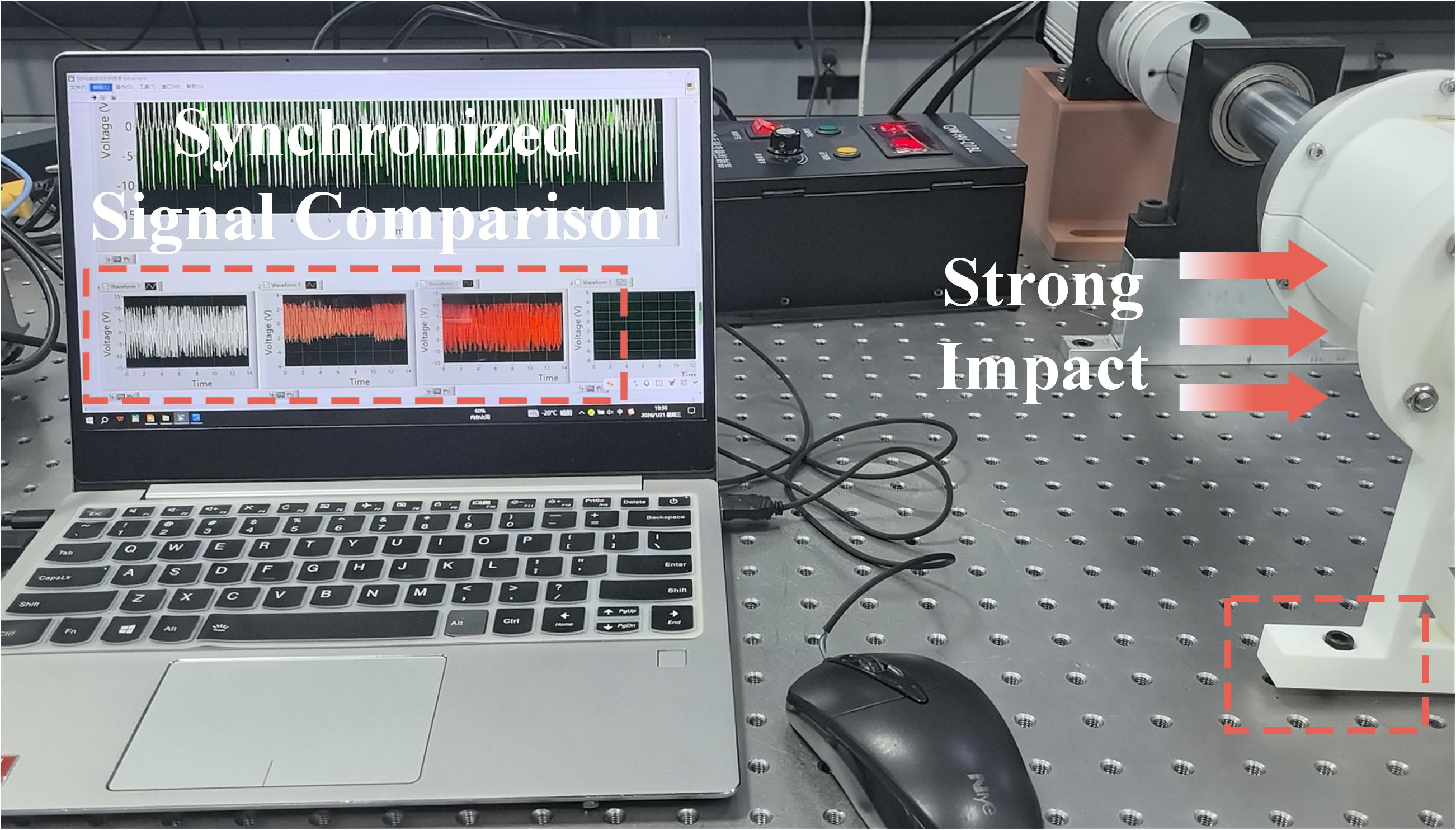


**Figure S3.** Simulating Strong Impact


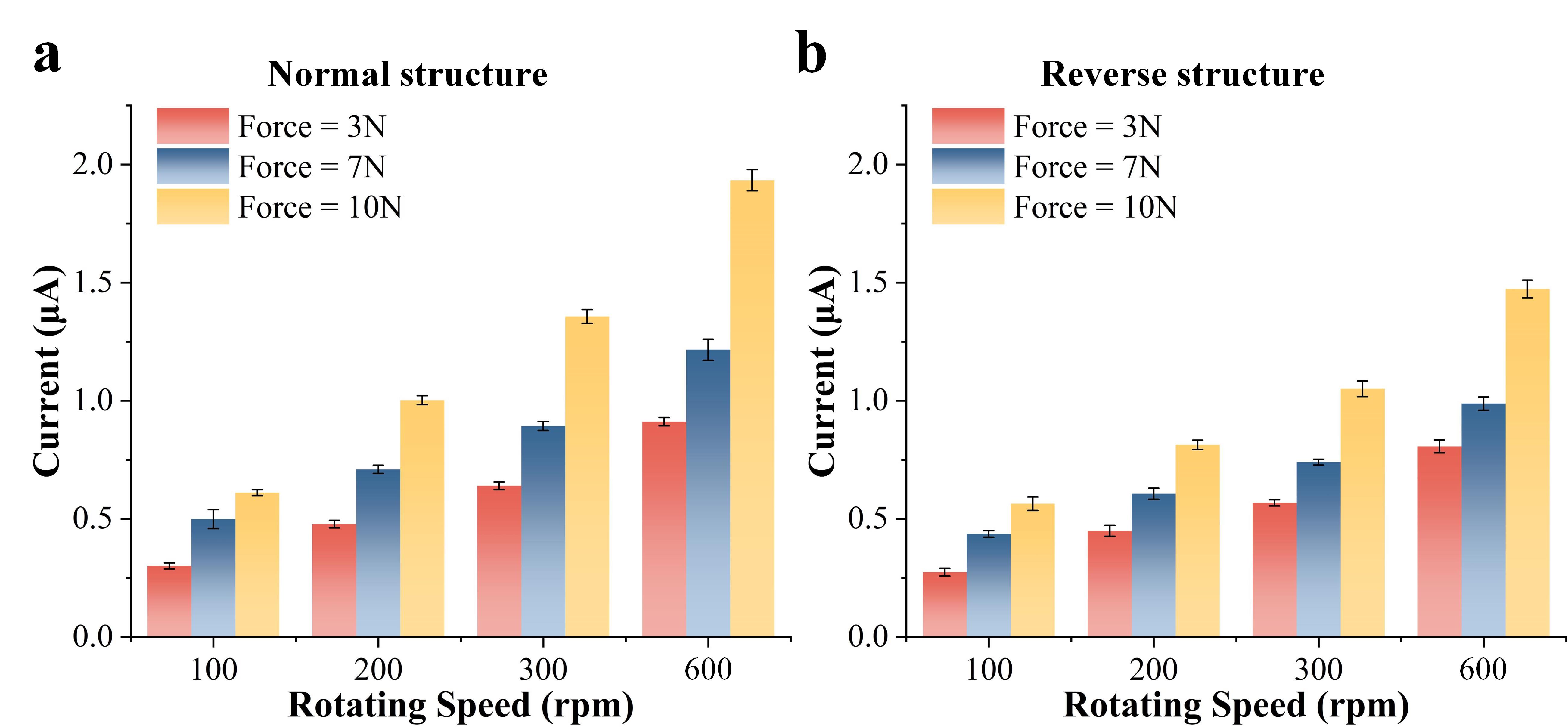


**Figure S4.** Comparison of current amplitude


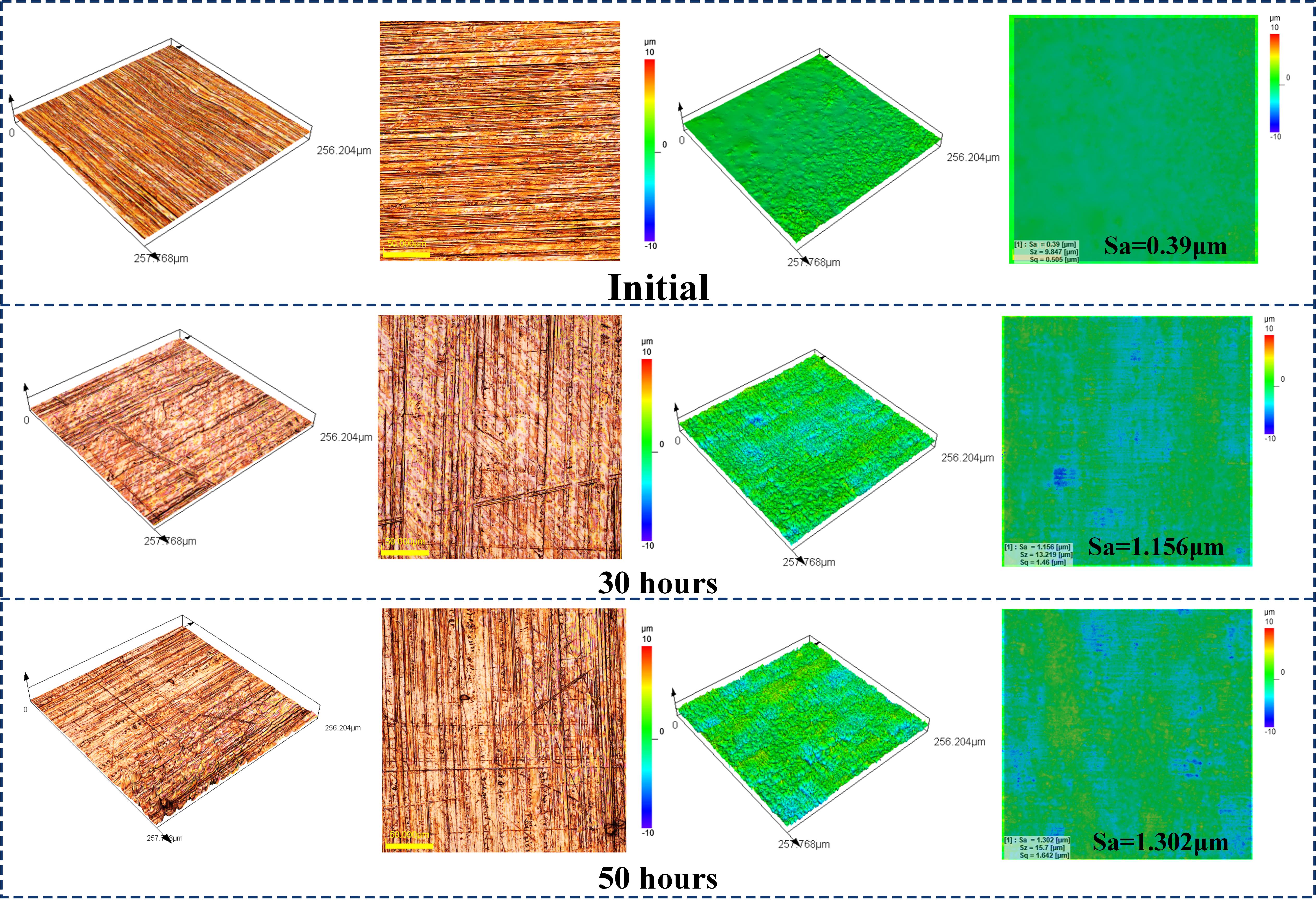


**Figure S5.** Surface morphology of PTFE and copper foil at different wear stages


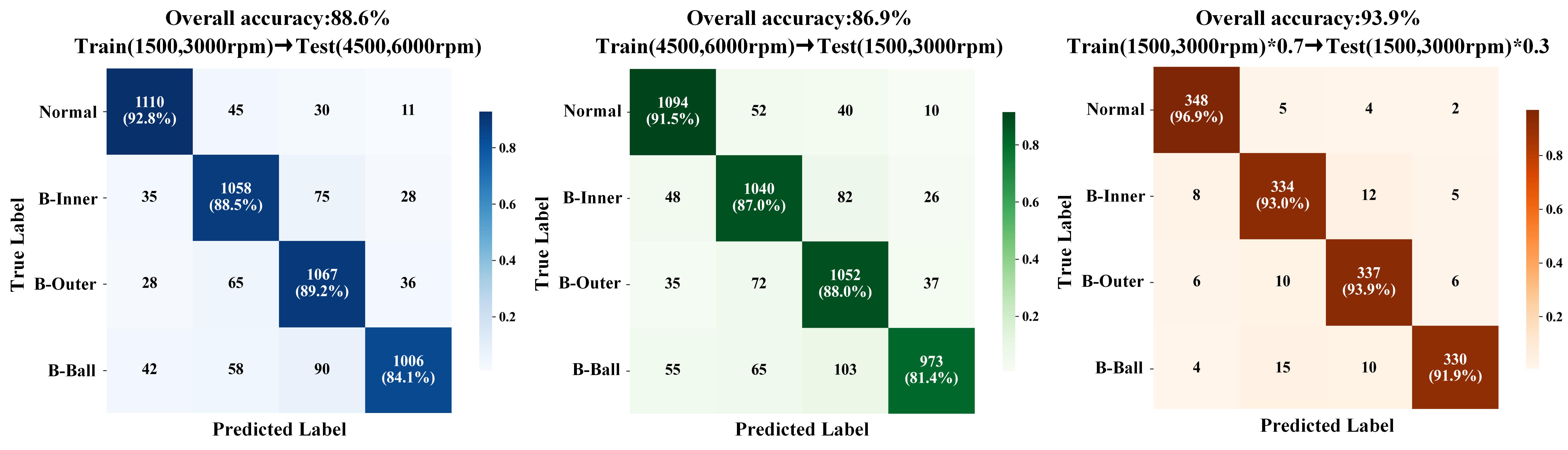


**Figure S6.** Cross validation confusion matrix comparison


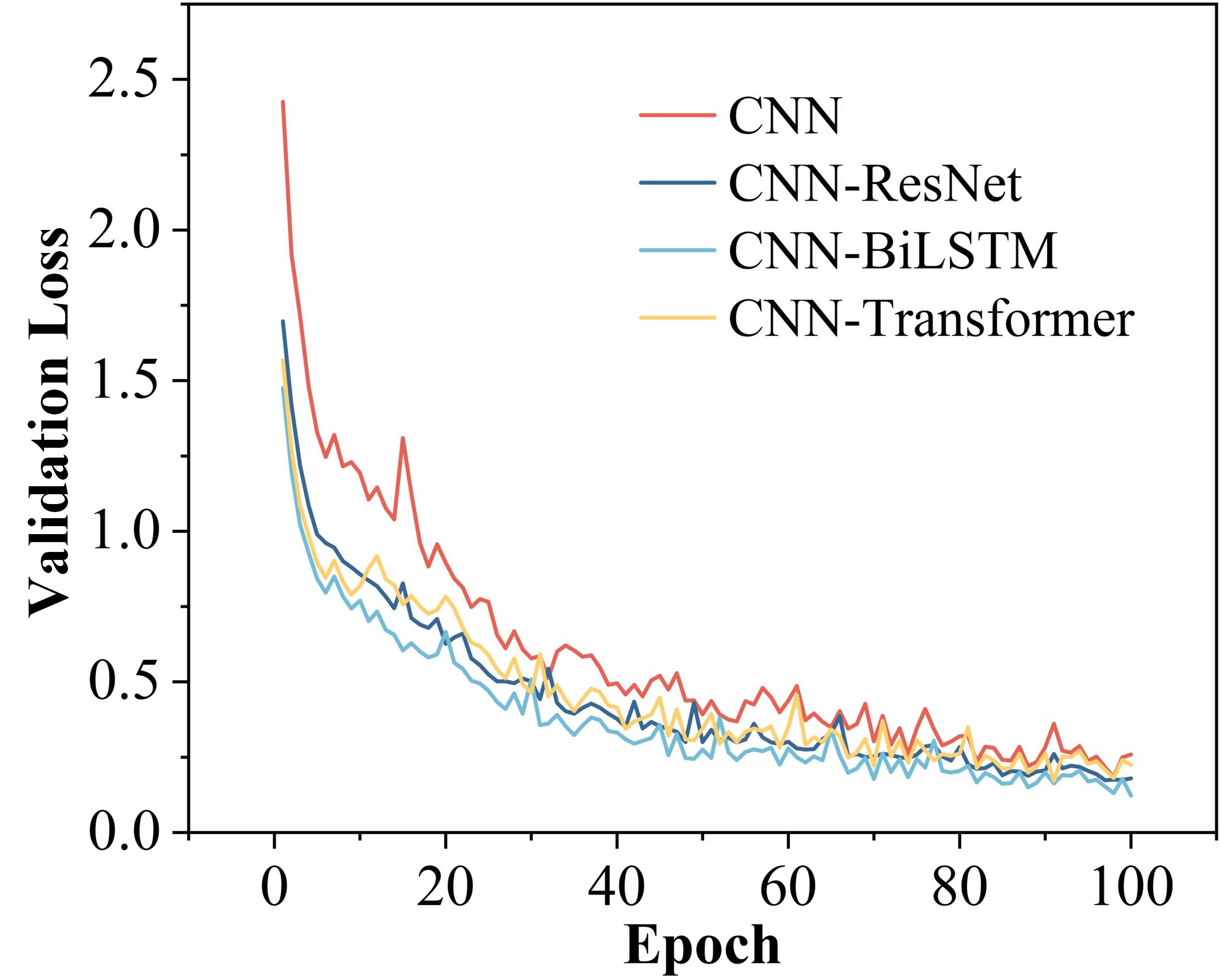


**Figure S7.** Comparison of Verification Losses


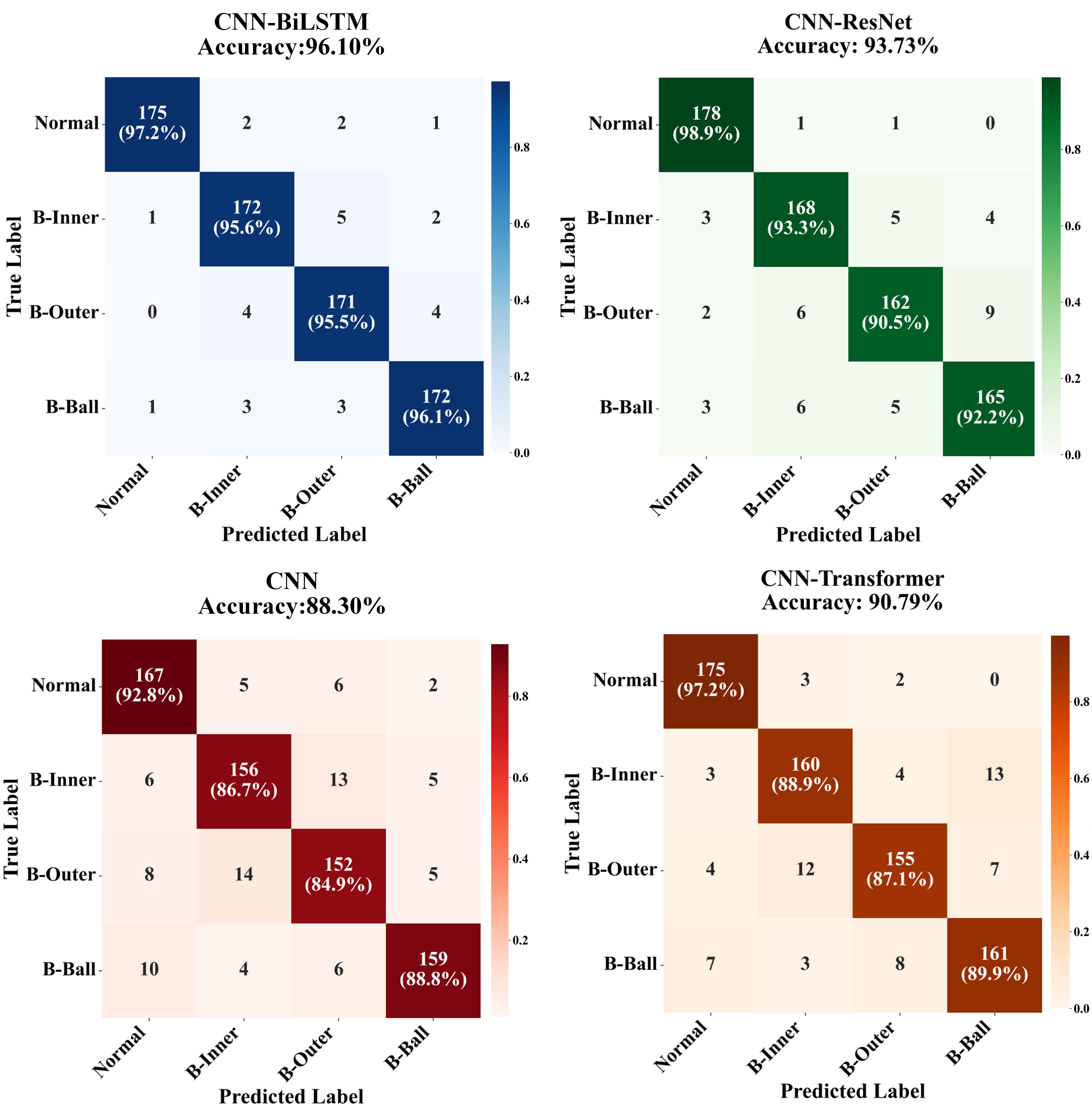


**Figure S8.** Comparison of confusion matrices for different models


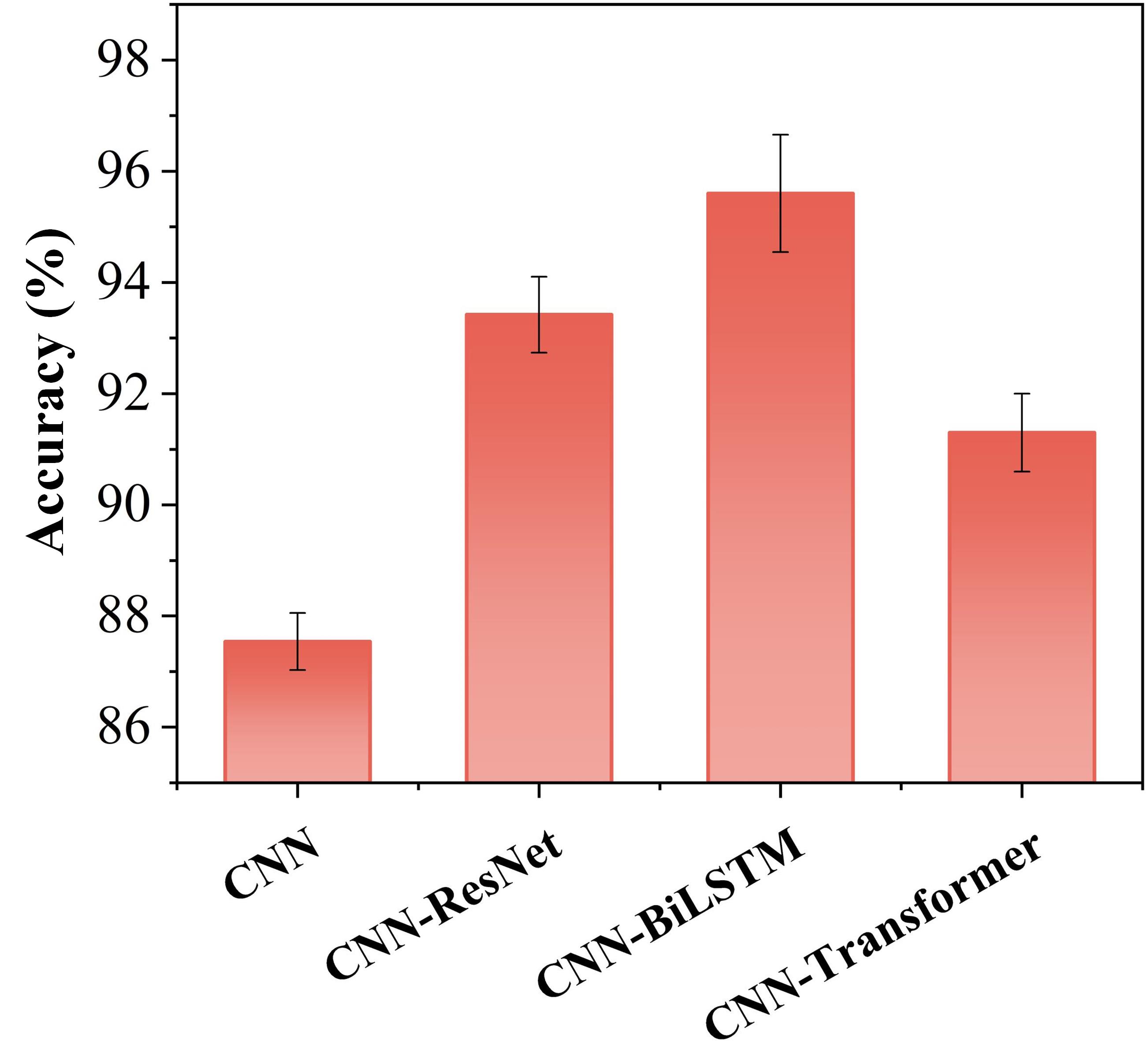


**Figure S9.** Comparison of Average Accuracy
